# Effect of Clozapine on DNA Methylation in Peripheral Leukocytes from Patients with Treatment-Resistant Schizophrenia

**DOI:** 10.3390/ijms18030632

**Published:** 2017-03-14

**Authors:** Makoto Kinoshita, Shusuke Numata, Atsushi Tajima, Hidenaga Yamamori, Yuka Yasuda, Michiko Fujimoto, Shinya Watanabe, Hidehiro Umehara, Shinji Shimodera, Takanobu Nakazawa, Masataka Kikuchi, Akihiro Nakaya, Hitoshi Hashimoto, Issei Imoto, Ryota Hashimoto, Tetsuro Ohmori

**Affiliations:** 1 Department of Psychiatry, Graduate School of Biomedical Sciences, Tokushima University, 3-8-15, Kuramoto-cho, Tokushima 770-8503, Japan; knst5511mkt@sunny.ocn.ne.jp (M.K.); wata-shin@tokushima-u.ac.jp (S.W.); umeharatokushima@yahoo.co.jp (H.U.); ohmori@tokushima-u.ac.jp (T.O.); 2 Department of Human Genetics, Graduate School of Biomedical Sciences, Tokushima University, Tokushima University Graduate School, 3-18-15, Kuramoto-cho, Tokushima 770-8503, Japan; atajima@med.kanazawa-u.ac.jp (A.T.); issehgen@tokushima-u.ac.jp (I.I.); 3 Department of Bioinformatics and Genomics, Graduate School of Advanced Preventive Medical Sciences, Kanazawa University, 13-1, Takaramachi, Kanazawa, Ishikawa 920-8640, Japan; 4 Department of Psychiatry, Graduate School of Medicine, Osaka University, D3, 2-2, Yamadaoka, Suita, Osaka 565-0871, Japan; yamamori@psy.med.osaka-u.ac.jp (H.Y.); yasuda@psy.med.osaka-u.ac.jp (Y.Y.); mfujimoto@psy.med.osaka-u.ac.jp (M.F.); hashimor@psy.med.osaka-u.ac.jp (R.H.); 5 Department of Neuropsychiatry, Kochi Medical School, Kohasu, Oko-cho, Nankoku, Kochi 783-8505, Japan; shimodes@kochi-u.ac.jp; 6 Department of Pharmacology, Graduate School of Dentistry, Osaka University, 1-8, Yamada-Oka, Suita, Osaka 565-0871, Japan; nakazawa@phs.osaka-u.ac.jp; 7 iPS Cell-based Research Project on Brain Neuropharmacology and Toxicology, Graduate School of Pharmaceutical Sciences, Osaka University, 1-6, Yamadaoka, Suita, Osaka 565-0871, Japan; hasimoto@phs.osaka-u.ac.jp; 8 Department of Genome Informatics, Graduate School of Medicine, Osaka University, 2-2, Yamadaoka, Suita, Osaka 565-0871, Japan; kikuchi@gi.med.osaka-u.ac.jp (M.K.); nakaya@gi.med.osaka-u.ac.jp (A.N.); 9 Molecular Research Center for Children’s Mental Development, United Graduate School of Child Development, Osaka University, D3, 2-2, Yamadaoka, Suita, Osaka 565-0871, Japan; 10 Laboratory of Molecular Neuropharmacology, Graduate School of Pharmaceutical Sciences, Osaka University, 1-6, Yamadaoka, Suita, Osaka 565-0871, Japan; 11 Division of Bioscience, Institute for Datability Science, Osaka University, 1-1 Yamadaoka, Suita, Osaka 565-0871, Japan

**Keywords:** schizophrenia, DNA methylation, clozapine

## Abstract

Clozapine is an atypical antipsychotic, that is established as the treatment of choice for treatment-resistant schizophrenia (SCZ). To date, no study investigating comprehensive DNA methylation changes in SCZ patients treated with chronic clozapine has been reported. The purpose of the present study is to reveal the effects of clozapine on DNA methylation in treatment-resistant SCZ. We conducted a genome-wide DNA methylation profiling in peripheral leukocytes (485,764 CpG dinucleotides) from treatment-resistant SCZ patients treated with clozapine (*n* = 21) in a longitudinal study. Significant changes in DNA methylation were observed at 29,134 sites after one year of treatment with clozapine, and these genes were enriched for “cell substrate adhesion” and “cell matrix adhesion” gene ontology (GO) terms. Furthermore, DNA methylation changes in the *CREBBP* (CREB binding protein) gene were significantly correlated with the clinical improvements. Our findings provide insights into the action of clozapine in treatment-resistant SCZ.

## 1. Introduction

Schizophrenia (SCZ) is a mental disorder characterized by symptoms that include delusions, hallucinations, and disorganized speech [1]. Approximately, 30% of SCZ patients are treatment-resistant [2], and the atypical antipsychotic clozapine has become the treatment of choice in this setting [3,4]. Recently, we have demonstrated that cell adhesion molecules, which play an important role in brain development including in axonal/dendrite growth, synapse formation and plasticity [5], were potential candidates for the molecular basis of clozapine response by conducting gene expression profiling using induced pluripotent stem (iPS) cell-based technology [6]. However, the molecular and epigenetic mechanisms underlying the therapeutic efficacy of clozapine have not yet been fully elucidated.

DNA methylation is an epigenetic modification that plays a critical role in brain function [7,8]. DNA methylation mainly occurs at the 5′ position of the cytosine base followed by a guanine base that is called CpG [9]. A number of studies have demonstrated aberrant DNA methylation in SCZ [10,11,12,13,14,15,16,17,18]. Furthermore, growing evidence suggests that DNA methylation may be involved in the therapeutic efficacy of atypical antipsychotic drugs [19,20,21,22,23,24]. With respect to clozapine, two animal studies have demonstrated that acute clozapine treatment induces DNA demethylation in the promoters of specific GABAergic and glutamatergic genes [25,26]. However, to our knowledge, no study investigating comprehensive DNA methylation changes in SCZ patients treated with chronic clozapine has been reported.

In the present study, we comprehensively analyzed changes in DNA methylation in peripheral leukocytes from treatment-resistant SCZ patients treated with clozapine in a longitudinal study. Next, we examined the correlation between changes in DNA methylation in response to clozapine and clinical improvements in treatment-resistant SCZ.

## 2. Results

### 2.1. Changes in DNA Methylation in Leukocytes after Clozapine Treatment

Of the 350,142 CpG sites analyzed, significant changes in DNA methylation were observed at 29,134 sites when we compared samples from 21 patients collected before and after one year of treatment with clozapine. The top 100 CpG sites are shown in Supplementary Table 1. Of the 29,134 CpG sites showing significant differences in methylation, clozapine treatment increased DNA methylation at 13,052 sites (44.8%) and decreased DNA methylation at 16,082 sites (55.2%).

**Figure 1:**
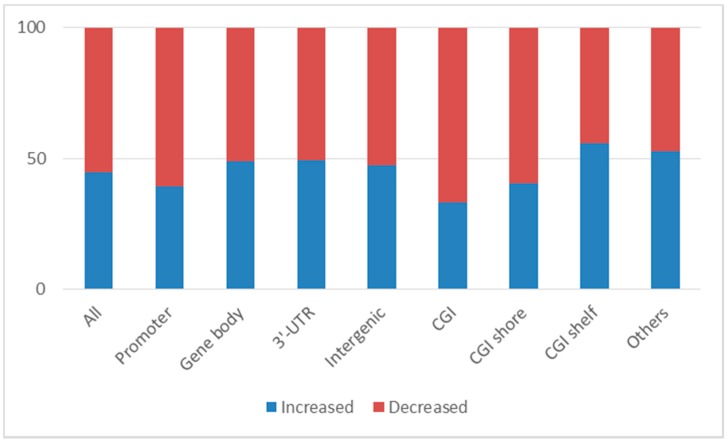
The proportions of CpG sites which showed increased or decreased DNA methylation changes after clozapine treatment. Of 29,134 significant CpG sites, clozapine caused an increased DNA methylation at 13,052 sites (44.8%) and decreased DNA methylation at 16,082 sites (55.2%). Of 29,134 significant CpG sites, 11,850 sites (40.7%) were located in the promoter regions (increased DNA methylation: 39.5%, decreased DNA methylation: 60.5%), 9479 sites (32.5%) in gene bodies (increased DNA methylation: 49.1%, decreased DNA methylation: 50.9%), and 864 sites (3.0%) in 3′-UTRs (increased DNA methylation: 47.5%, decreased DNA methylation: 52.5%). Of 29,134 CpG sites, 7656 sites (26.3%) were located in the CGIs (CpG island) (increased DNA methylation: 33.1%, decreased DNA methylation: 66.9%), 7334 sites (25.2%) in CGI shores (increased DNA methylation: 40.3%, decreased DNA methylation: 59.7%), and 2846 sites (9.8%) in CGI shelves (increased DNA methylation: 55.9%, decreased DNA methylation: 44.1%).

Classification of significant CpG sites based on their locations within genes revealed that 11,850 sites (40.7%) were located in promoter regions; 9479 sites (32.5%) were in gene bodies; 864 sites (3.0%) were in 3′-UTRs; the remainders were found in intergenic regions. Decreases in DNA methylation following clozapine treatment were more likely to occur in promoter regions than in other regions (60.5% in promoter regions vs. 51.6% in other regions; Fisher’s exact test *p* = 7.00 × 10^−16^; Figure 1). Classification of CpG sites based on location relative to CpG content in the genes (CpG island (CGI), CGI shore, CGI shelf, and others) revealed that 7656 sites (26.3%) were located within CGIs; 7334 sites (25.2%) were in CGI shores; and 2846 sites (9.8%) were in CGI shelves. Sites of decreased DNA methylation following clozapine treatment were more likely to occur in CGI regions than in other regions (66.9% in CGI regions versus 51.0% in other regions; Fisher’s exact test *p* = 3.83 × 10^−36^; Figure 1). Interestingly, significant CpG sites of decreased DNA methylation which were located in CGIs in promoter regions include several GABA, glutamate, and related SCZ susceptibility genes [27], such as GAD1 (glutamate decarboxylase 1), GRIN2A (glutamate ionotropic receptor NMDA type subunit 2A), GRIN2D (glutamate ionotropic receptor NMDA type subunit 2D), and GRM7 (glutamate metabotropic receptor 7).

**Figure 2:**
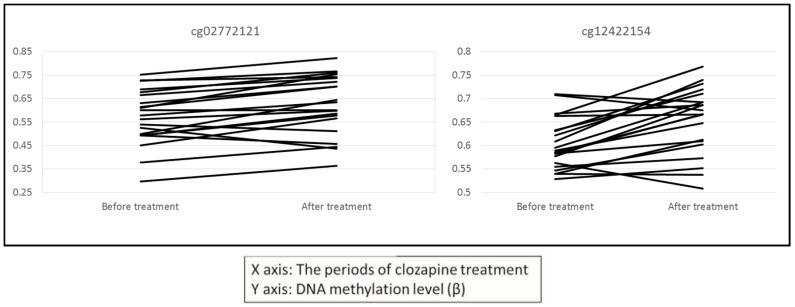
DNA methylation changes following clozapine treatment in the *TRIM15* gene. Clozapine caused increased DNA methylation changes at two CpG sites (cg02772121 and cg12422154) in the promoter region of the *TRIM15* gene (*p* = 3.2 × 10^−4^, and *p* = 2.5 × 10^−4^, respectively).

A list of the CpG sites located in promoter regions with average DNA methylation differences (Δβ) greater than 0.05 and paired *t*-test *p*-values less than 0.001 is shown in Table 1. Two of these CpG sites are located in the *TRIM15* (tripartite motif containing 15) gene, which has been implicated in SCZ (Figure 2) [28]. Additionally, gene ontology (GO) analysis revealed that genes with DNA methylation changes following clozapine treatment were enriched for the “cell substrate adhesion” and “cell matrix adhesion” GO terms (False discovery rate (FDR) *q* < 0.05).

### 2.2. Correlations between Changes in DNA Methylation in Leukocytes and Clinical Outcomes

**Figure 3:**
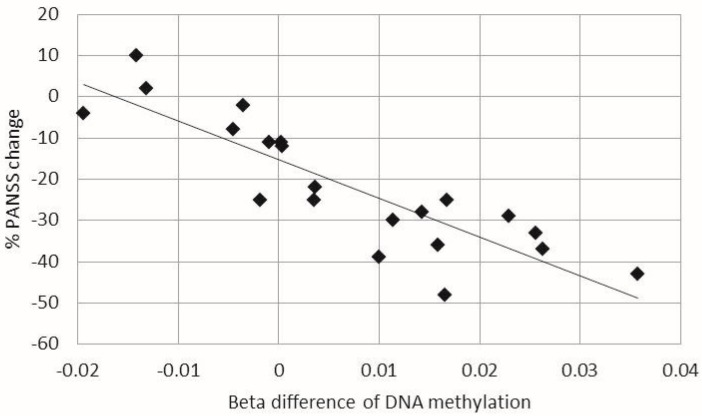
The correlation between beta difference of DNA methylation changes in the *CREBBP* gene and % Positive and Negative Syndrome Scale (PANSS) changes. X axis represents beta difference of DNA methylation in the *CREBBP* gene (cg05151055). Y axis represents % PANSS changes. Each dot represents samples. Significant negative correlation between beta difference of DNA methylation in the *CREBBP* gene and % PANSS changes was observed (*p* = 2.7 × 10^−7^).

Analysis of correlations between changes in psychotic symptoms (% Positive and Negative Syndrome Scale (PANSS)) and clinical variables (clozapine dose, duration of clozapine treatment, age of onset, gender, and use of mood stabilizers) revealed no significant correlations (*p* > 0.05). Upon analysis of correlations between ∆β-values for the 29,134 CpG sites showing significant changes in DNA methylation after clozapine treatment and % PANSS, a CpG site associated with the *CREBBP* (CREB binding protein) gene, cg05151055, was the only site that was significantly correlated with changes in psychotic symptoms (FDR *q* < 0.05; Figure 3).

## 3. Discussion

To the best of our knowledge, this study represents the first comprehensive analysis of the effects of clozapine treatment on DNA methylation in leukocytes from treatment-resistant SCZ patients. We identified 29,134 CpG sites that showed significant changes in DNA methylation following chronic clozapine treatment. The proportion of CpG sites with decreased DNA methylation was higher than the proportion of sites with increased DNA methylation after clozapine treatment (55.2% vs. 44.8%, respectively). Consistent with this finding, we previously demonstrated that DNA hyper-methylation patterns frequently occurred in medication-free patients with SCZ, while hypo-methylation was more common in patients with SCZ treated with antipsychotics [12,14]. We also found that decreased DNA methylation following clozapine treatment was more likely to occur at CpG sites located in CGIs in gene promoter regions. This trend is consistent with results reported in a previous study of the effects of blonanserin, another atypical antipsychotic [22].

In this study, we found that genes that showed DNA methylation changes following clozapine treatment were enriched for the GO terms “cell substrate adhesion”, which is essential for cells to interact with their environments [29], and “cell matrix adhesion”, which is essential for cell migration, tissues organization, and differentiation [30]. Similarly, in a recent analysis of differential gene expression profiles in neurons from twins with treatment-resistant SCZ who had discordant responses to clozapine using pluripotent stem (iPS) cell based technology, we found that differentially expressed genes were enriched for “cell adhesion” and “biological adhesion” [6]. Cell adhesion is essential for forming tissue and neuronal connections crucial for nervous system development [31]. Animal studies suggest that dysfunction of neuronal cell adhesion molecules, which are expressed primarily in the central nervous system where they regulate synaptic signaling, may lead to impairment of memory and learning [32,33]. Furthermore, pathways related to cell adhesion have been shown to contribute to SCZ susceptibility in a genome-wide association study [34], and increased blood levels of neuronal cell adhesion molecules have been observed in SCZ [35,36]. These results suggest that changes of neuronal cell adhesion-related molecules may be implicated in the pathophysiology of SCZ, and clozapine may exert its therapeutic effects by altering DNA methylation of genes encoding these molecules.

We found that increases in DNA methylation of the *CREBBP* gene following clozapine treatment was significantly correlated with clinical improvements in treatment-resistant SCZ. This result suggests that epigenetic modification of the *CREBBP* gene in peripheral leukocytes can predict clinical responses to clozapine in treatment-resistant SCZ. CREBBP is a protein that possesses intrinsic histone acetyltransferase activity, in addition to acting as a scaffold to stabilize protein interactions within the transcription complex [37]. Pathway analyses of results from genome-wide association studies have demonstrated that this gene is associated with SCZ [38,39]. Furthermore, *CREBBP* variants have been associated not only with clinical symptoms, but also with cognitive phenotypes in SCZ [36,40].

Our study has several limitations that should be noted. First, our sample size was small, and studies that replicate our findings in a larger cohort are needed. Second, patients enrolled in this study had been treated with various kinds of antipsychotics prior to treatment with clozapine. Third, the dosage and duration of clozapine treatment were not uniform across the patient cohort. Fourth, it is unclear whether changes in DNA methylation following clozapine treatment are normalized to control levels due to a lack of control data. Fifth, it is also unclear whether the DNA methylation changes following the treatment are specific to clozapine or are common among other antipsychotics. Direct comparisons to previous studies that examined the effects of antipsychotics on DNA methylation are hampered by the use of different study designs and analysis of different tissues [19,20,21,22,23,24]. Further blood studies using other antipsychotics are needed to reveal differences between clozapine and other antipsychotics. Sixth, we did not investigate the relationship between DNA methylation and expression. Further functional studies, including transcriptome analysis and cell adhesion assays, are required to clarify the molecular mechanisms of clozapine action. Finally, it is important to note that the observed changes in DNA methylation after clozapine treatment were analyzed only in peripheral leukocytes, and studies in brain tissue are needed to confirm the mechanistic interpretation of our results.

## 4. Materials and Methods

### 4.1. Subjects

Twenty-one patients with SCZ (mean age: 42.1 ± 11.4 years; eight males and 13 females) were recruited from Kochi, Tokushima, and Osaka University Hospitals in Japan. All subjects were of Japanese origin and had been treated with various antipsychotic drugs before treatment with clozapine. Peripheral blood was collected twice from each patient, just before introduction of clozapine and after one year of treatment. The mean dose of clozapine was 473.8 ± 91.3 mg/day, and the mean duration of clozapine treatment was 340.8 ± 182.7 days. The psychotic symptoms of the patients were evaluated using PANSS at the time of peripheral blood sample collection. The mean PANSS score was 113.4 at baseline and 91.7 at the end of treatment, respectively. The clinical characteristics of the patients are summarized in Supplementary Table 2. SCZ was diagnosed according to DSM-IV criteria by at least two expert psychiatrists on the basis of extensive clinical interviews and a review of medical records. All patients met the criteria for treatment-resistant SCZ and clozapine administration, as described in the clozapine drug information provided in Japan [41,42]. No psychiatric comorbidities were present in any of the patients. The study protocol was approved by the institutional ethics committee of Tokushima University Graduate School (Project ID, H28-1; Date of approval, 23 June 2016), and all enrolled participants provided their signed, written, informed consent for participation.

### 4.2. Analysis of DNA Methylation

Genomic DNA was extracted from peripheral blood using the QIAamp DNA Blood Mini Kit (Qiagen, Germantown, MD, USA). Bisulfite conversion of 500 ng of genomic DNA was performed with the EZ DNA methylation kit (Zymo Research, Irvine, CA, USA). DNA methylation levels were assessed using Infinium^®^ HumanMethylation450 BeadChips (Illumina Inc., San Diego, CA, USA), which makes it possible to examine DNA methylation status at more than 485,000 CpG sites, according to the manufacturer’s instructions, and the resulting data was analyzed using the methylation analysis module within the BeadStudio software (Illumina Inc.). The DNA methylation status of CpG sites (termed the β-value) was calculated based on the ratio of the signal from a methylated probe relative to the sum of the signal from both methylated and unmethylated probes. β values ranged from 0 (completely unmethylated) to 1 (fully methylated). For intra-chip normalization of probe intensities, colored balance and background corrections were performed for every set of 12 samples from the same chip using internal control probes. CpG sites used for statistical analyses met the following criteria: (1) β-values with detection *p*-values < 0.05; (2) autosomal CpGs, with no missing values in any subjects; (3) no probe single nucleotide polymorphisms (SNPs) with minor allele frequencies ≥5% in the HapMap-JPT population; (4) no probe cross-reactivity; and (5) no SNPs at CpG sites and single-base extension sites detailed in a previous paper [43]. The final data set for peripheral leukocytes included 350,142 sites.

### 4.3. Statistical Analysis

A paired *t*-test was used to assess DNA methylation changes following clozapine treatment. *p*-values < 0.05 were considered statistically significant. Gene ontology analysis was performed using the Database for Annotation, Visualization, and Integrated Discovery (DAVID) [44]. An FDR correction was applied at the 0.05 level for multiple comparisons. Pearson’s correlations were performed to assess the effects of clinical variables, including clozapine dose, duration of clozapine treatment, age of onset, gender, and use of mood stabilizers on PANSS percentage changes (% PANSS; defined as (PANSS at the end of the study − PANSS at baseline)/PANSS at baseline). A univariate linear regression model was used to examine the relationship between β-values for probes with significant DNA methylation changes following clozapine treatment and % PANSS. An FDR correction was applied at the 0.05 level for multiple comparisons.

## 5. Conclusions

We report a comprehensive analysis of DNA methylation changes in peripheral leukocytes from patients with treatment-resistant SCZ following clozapine treatment. We found that genes with clozapine-induced changes in DNA methylation were associated with cell substrate adhesion and cell matrix adhesion. These results provide insight into potential mechanisms of action of clozapine in treatment-resistant SCZ. Further functional studies are needed to clarify the molecular mechanisms of clozapine action.

## Figures and Tables

**Table 1:** A list of the significant CpG sites with average Δβ > 0.05 and paired *t*-test *p*-value < 0.001 in the gene promoter regions. CGI, CpG island; UCSC, University of California Santa Cruz; * Positions refer to Genome Research Consortium human genome build 37 (GRCh37/UCSC human genome 19 (hg19).

Probe ID	Average Beta Difference before Treatment of Clozapine	Average Beta Difference after Treatment of Clozapine	Average Beta Difference between Treatment of Clozapine	*p*-Value	Chromosome	Position *	UCSC RefGene Name	UCSC RefGene Group	Relation to UCSC CpG Island
cg15542713	0.427	0.496	0.070	1.1 × 10^−4^	1	42385581	*HIVEP3*	Promoter	CGI shore
cg02772121	0.570	0.624	0.054	3.2 × 10^−4^	6	30130881	*TRIM15*	Promoter	Others
cg10864200	0.608	0.557	−0.050	7.3 × 10^−4^	4	720809	*PCGF3*	Promoter	CGI shelf
cg12422154	0.601	0.652	0.050	2.5 × 10^−4^	6	30130819	*TRIM15*	Promoter	Others

## Supplementary Materials

Supplementary materials can be found at www.mdpi.com/1422-0067/18/3/632/s1.
