# Supplementary material for: Effect of Clozapine on DNA Methylation in Peripheral Leukocytes from Patients with Treatment-Resistant Schizophrenia
Source: Int J Mol Sci. 2017 Mar 14;18(3):632. doi: 10.3390/ijms18030632 (PMC5372645; doi:10.3390/ijms18030632)
Supplement: Supplementary file 1 [file ijms-18-00632-s001.zip › ijms-178454-supplemetary Table S2.docx]

Supplementaly Table 2. The clinical characteristics of the patients

| Number of samples (male/female) | 21 (8/13) |
| --- | --- |
| Mean age (SD) | 42.1 y.o. (11.4) |
| Mean age at onset (SD) | 24.0 y.o. (8.6) |
| Mean duration of clozapine treatment (SD) | 340.8 days (182.7) |
| Mean clozapine dose (SD) | 473.8 mg/day (91.3) |
| Mean PANSS at baseline (SD) | 113.4 (25.0) |
| Mean PANSS at the end of the study (SD) | 91.7 (18.4) |
